# Aortic valve surgery: management and outcomes in the paediatric population

**DOI:** 10.1007/s00431-021-04092-1

**Published:** 2021-05-10

**Authors:** Mariam Zaidi, Ganeshkumar Premkumar, Rimel Naqvi, Arwa Khashkhusha, Zahra Aslam, Adil Ali, Abdulla Tarmahomed, Amr Ashry, Amer Harky

**Affiliations:** 1 Charing Cross Hospital, Imperial College NHS Trust, Fulham Palace Rd, Hammersmith, London, W6 8RF UK; 2 St George’s University of London, Cranmer Terrace, Tooting, London, SW17 0RE UK; 3 University of Liverpool, School of Medicine, Liverpool, L69 3BXl UK; 4 Faculty of Medicine, Imperial College London, Exhibition Rd, South Kensington, London, SW7 2BU UK; 5 Department of Paediatric Cardiology, Alder Hey Children’s Hospital, Liverpool, UK; 6 Department of Paediatric Cardiac Surgery, Alder Hey Children’s Hospital, Liverpool, UK; 7 Department of Cardiothoracic Surgery, Assiut University Hospital, Asyut, Egypt; 8 Department of Cardiothoracic Surgery, Liverpool Heart and Chest Hospital, Liverpool, UK

**Keywords:** Paediatric, Aorta, Congenital anomaly, Surgery

## Abstract

Congenital anomalies of the aortic valve frequently necessitate intervention in childhood. The most common aortic valve pathologies present in childhood are aortic stenosis and insufficiency. Presentation of aortic valve disease depends on severity and presence of concomitant syndromes and valvular disorders. Treatment options are largely categorised as medical, percutaneous repair or surgical repair and replacement. Surgical techniques have been refined over the last few years making this the mainstay of treatment in paediatric cases. Whilst repair is considered in most instances before replacement, there are substantial limitations which are reflected in the frequency of reintervention and restenosis rate. Replacements are typically undertaken with tissue or mechanical prosthesis. The current gold-standard aortic valve replacement surgery is called the Ross procedure—where replacement is undertaken with a competent pulmonic valve and a simultaneous pulmonary homograft.

*Conclusion*: In this review, we aim to outline the various surgical options and discuss efficacy and complications of various interventions.

**Table:** 

**What is Known:** • *Congenital aortic valve defects repair options medically and surgically*	
**What is New:** • *Comparisons between surgical options for aortic valve repair including efficacy, risks and long-term outcomes*.

**What is Known:**

• *Congenital aortic valve defects repair options medically and surgically*

**What is New:**

• *Comparisons between surgical options for aortic valve repair including efficacy, risks and long-term outcomes*.

## Introduction

**Fig. 1:**
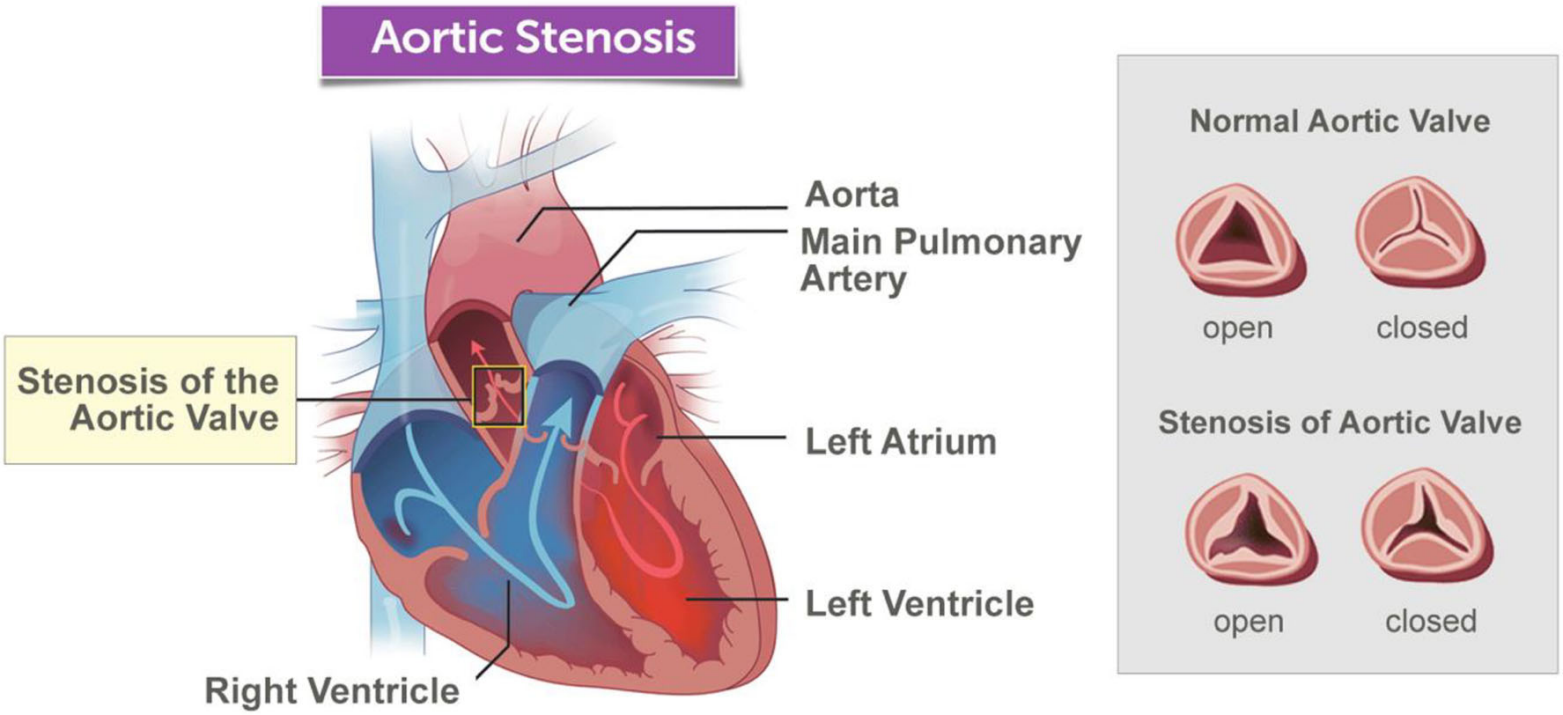
Anatomy of the aortic valve (https://www.childrenshospital.org/conditions-and-treatments/conditions/a/aortic-valve-stenosis)

A normal aortic valve comprises a tri-leaflet structure situated between the left ventricular outflow tract and aortic root (Fig. 1). Within the paediatric population, various pathologies can lead to an impairment of aortic valve function. Such pathologies include aortic valvular atresia, aortic valve stenosis (AVS) and bicuspid aortic valve. Aortic valve atresia (AVA) is a congenital condition in which the cusps of the aortic valve are fused at birth and commonly occurs as part of a range of abnormalities of the left ventricular outflow tract (LVOT). AVS occurs as a result of a complicated pathogenic process, which can be described as altered mechanical stress exerted upon the valves, followed by subsequent valvular inflammation, fibrosis and leaflet thickening with calcification [1]. The stiffening of the aortic valves leads to a reduction in the lumen opening for blood flow. Aortic valve regurgitation, otherwise termed insufficiency, occurs when there is backflow of blood from the aorta into the left ventricle. This can be due to many factors including aortic valve dysfunction such as that seen in aortic valve stenosis [2].

The most common presentation of AVA is aortic stenosis (AS); however, in rare cases, complete atresia can be present [3]. A congenital bicuspid aortic valve has two functional leaflets present instead of three and may be detectable in 1–2% of all paediatric congenital heart disease. It has been shown to be the primary cause of aortic stenosis in 70–85% of paediatric cases and predisposes the aortic valve to rheumatic heart disease [3, 4]. Another possible complication of the bicuspid aortic valve and less commonly rheumatic heart disease is aortic valve regurgitation (AVR), which occurs due to the inability of the aortic valve leaflets to remain closed during diastole, resulting in retrograde blood flow and an overall increase in end-diastolic volume leading to elevation in wall stress [5, 6].

The most common impairment that results from such pathologies is aortic valve stenosis (AVS) which accounts for nearly 3–6% of all congenital heart defects. Within the paediatric population, AVS is virtually always congenital in origin. AVS in the foetal and infant population is associated with reduced development of left-sided heart structures (hypoplastic left heart syndrome) and left ventricular endocardial fibroelastosis and dysfunction [7]. The pathophysiology of AVS follows a path of valvular inflammation, fibrosis and valve thickening, resulting in valvular calcification and outflow obstruction. Valvular inflammation may arise for a number of reasons including abnormal blood flow through a bicuspid aortic valve or even the inflammatory process associated with rheumatic heart disease. In recent times, a genetic basis for the underlying pathogenesis of AVS has been identified. One study found significant alterations in CpG methylation at 59 sites in 52 genes in patients with AVS; these genes were found to be involved in positive regulation of receptor-mediated endocytosis [8].

Aortic valve diseases are one of the most common causes of congenital cardiac disease, occurring in 1–2% of patients; it is difficult to estimate the incidence specifically in paediatric populations, as the age at presentation and diagnosis can vary greatly [9]. In isolated aortic valve disease, the condition is independent of any underlying pathology. However, aortic valve disease can arise from or give rise to many other pathologies; it is commonly associated with aortic regurgitation and infective endocarditis. Additionally, it is estimated that 50% of severe aortic stenosis cases present on a background of aortic valve disease. This clinical picture is reflected in patients with hypoplastic left heart syndrome [10]. An under-recognised congenital heart disease is shone complex, in which subaortic stenosis occurs due to membranous or muscular thickening forming below the aortic valve.

## Clinical presentation

The clinical presentation of a patient with an impaired aortic valve can vary greatly depending upon the type and severity of the impairment and whether there are any coexisting abnormalities. The degree of AS can be divided into critical and non-critical. Critical stenosis typically becomes symptomatic during the neonatal or early infancy period whereas non-critical stenosis may be asymptomatic until later in childhood [11]. These presentations are highlighted in Table 1.

**Table 1:** A comparison of the clinical presentation of critical vs non-critical aortic valve stenosis

Degree of stenosis	Critical	Non-critical
Symptom onset	Within days to weeks	Can be asymptomatic for many years
Cyanosis	Acyanotic	Acyanotic
Cardiac signs	Signs of congestive heart failure within first weeks of life	Exertional chest pain (childhood years)
Respiratory signs	Pulmonary congestion	Minimal
Left ventricle hypertrophy	Present	Present

The screening for cardiac abnormalities that occur as part of neonatal and developmental checks often serves poorly in the detection of severe AVS. Most infants with severe AVS will display symptoms of progressive heart failure by 2 months of age. They present as pale, hypotensive and dyspnoeic with approximately 50% having a normal first heart sound, and ejection click and a gallop. In some cases, a foetus may develop hypoplastic left heart syndrome (HLHS) as a result of AVS. AVS can also present as part of shone syndrome. In those who have a functioning atrial septum, the onset of cyanosis and respiratory distress will occur soon after birth. Those with an atrial septal defect may appear acyanotic at first but develop signs of systemic hypoperfusion, respiratory distress and lethargy following the closure of the patent ductus arteriosus [11]. The diagnosis of AVS in a foetus can be achieved through the use of foetal echocardiography showing a thickened or domed aortic valve with an increased Doppler velocity >1 m/s [6].

Children and adolescents with AVS tend to be asymptomatic and only around 10% of these children display symptoms such as angina or syncope predominantly during exercise [12]. Similarly, the majority of children with bicuspid aortic valve tend to be asymptomatic with those who experience symptoms noticing a mild reduction in exercise tolerance or more noticeable symptoms such as exertional angina, syncope and dizziness. The risk of developing AVS in children with isolated bicuspid aortic valve increases with age [13].

## Investigations

Timely investigation of aortic valve morphology and lesion severity is paramount in paediatric patients [14]. Imaging has a vital role in both diagnosis and risk stratification of aortic valvular pathology, as well as assessing left ventricular adequacy and function [15]. When performed appropriately, imaging can help inform the timing and necessity of valvular intervention, factors which are vital determinants of prognosis and clinical outcomes [12, 14].

### Echocardiography

**Fig. 2:**
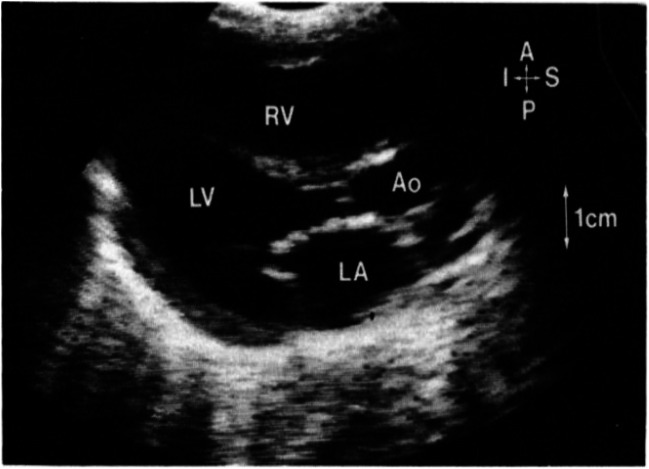
Parasternal long-axis view of neonatal critical aortic stenosis. A hypoplastic aortic valve annulus and post-stenotic dilation of the ascending aorta are noted

Echocardiography plays a vital role in the anatomical assessment and presence of aortic valve pathology in the paediatric patient population [12, 14]. It is safe, non-invasive, inexpensive and widely available, making it the imaging modality of choice in the effective evaluation of the paediatric aortic valve [12, 14]. Transthoracic echocardiography (TTE) is the gold-standard procedure [5, 14] in the diagnosis and follow-up of congenital aortopathy, such as valvular aortic stenosis (VAS) and bicuspid aortic valves (BAV) [12]. This form of image acquisition (as seen in Fig. 2) utilises ultrasound waves in order to obtain images of the myocardium and vascular structures [1]. An ultrasound machine with 2D mode, M-mode, colour flow Doppler mapping and CW Doppler is coupled with an appropriate transducer in order to obtain functional images [16]. Doppler velocity signals can then be used in order to determine the presence and severity of stenosis in the aortic valve, as well as allowing haemodynamic assessment [16–18]. Doppler echocardiography allows for analysis of peak and mean velocities through the aortic valve which can, in turn, be used to calculate mean pressure gradients using the modified Bernoulli formula and the aortic valve area (AVA) using the continuity equation [16]. These parameters are useful diagnostic indicators, especially in low-flow states. Information provided by peak velocity, mean gradient and AVA is combined in order to grade the haemodynamic severity of aortic stenosis [14]. Although the consensus is that this approach is effective in determining the extent of stenosis, the wide spectrum of diagnostic categories may create potential clinical confusion. Furthermore, in certain patients, perfect alignment of the Doppler probe with the direction of maximal blood flow through the aortic valve may not be possible which would result in problems acquiring diagnostic acoustic windows [14]. Images can also be obtained transoesophageally. However, this is difficult to facilitate at the bedside, as it has poor tolerance in paediatric patients [16, 19] and is mostly undertaken under sedation and/or anaesthesia, which carry their own risks.

There is an established role of echocardiography in the antenatal diagnosis of aortic valve pathologies and insufficiency. An example of one such pathology is congenital aortic stenosis (CAS) [20], which can lead to endocardial fibroelastosis, hypoplastic left heart syndrome and congestive heart failure [20, 21]. A neonate with CAS may require surgery in the first days to weeks of life in order to thrive. Echocardiogram allows for the antenatal diagnosis of CAS as early as 16 weeks gestational age, which enables clinicians to formulate more focused management plans and exercise early intervention. Families are then able to make better-informed decisions regarding the prospective outcomes in utero or in neonatal life. For instance, some cases of CAS, namely critical CAS, may not be compatible with life; echocardiogram allows this assessment to be made in a timely manner in order to aid reflections on possible termination of the pregnancy, invasive intervention in the immediate neonatal period or compassionate care [20, 21]. One can also predict the type of intervention that would be most likely required for a patient based on echocardiographic markers such as valvular annulus sizes, LV dimensions and volume, as well as septal thicknesses.

Nevertheless, certain pathologies of the aortic valve can be quite difficult to diagnose antenatally and present significant challenges to clinicians. Specifically, coarctation of the aorta is the most common ductal-dependent lesion which is missed on antenatal assessment [3] and can subsequently lead to rapid deterioration of a neonate. This is mainly due to findings on imaging being inconclusive [22]. Echocardiography provides the invaluable opportunity to closely study the aortic valve in the foetus and differentiate inconclusive anatomy suggesting the possibility of coarctation from clinical cases. This allows for fast-tracking of patients deemed to be eligible candidates for neonatal echocardiography, thus preventing as critical a pathology as coarctation from being missed in the neonate [22].

### Cardiac computed tomography (CCT)

This is an exceptional imaging tool which yields high-quality images and allows essential preoperative planning for transcatheter aortic valve replacement (TAVR) [23]. Although TAVR is not routinely used in the paediatric population, a study conducted in 2019 found this method of valve replacement to be a reasonable alternative to surgical aortic valve replacement in certain paediatric patients with suboptimal predicted surgical outcomes [24]. Furthermore, CCT may aid in characterising aortopathy in patients with inconclusive echocardiography findings [16]. A major drawback, nevertheless, is the inability to measure flow velocity or volume, a well-established component of echocardiography [14, 17].

### Cardiac magnetic resonance (CMR)

CMR and 4D flow MRI provide robust data on patient-specific aortic haemodynamics [24–26]. This helps to predict risk of disease progression, particularly in BAV20. The temporal resolution of this modality depends on pulse sequence and heart rate, unlike echocardiography [17].

CMR is particularly advantageous due to the opportunity it offers to detect concomitant pathologies. Diseases such as myocarditis which present significant morbidity and mortality risks in paediatric patients can be non-invasively diagnosed using CMR, preventing the necessity for invasive procedures such as endomyocardial biopsies [27].

CMR provides a higher degree of sensitivity to congenital heart disease than echocardiography and catheterisation, owing to its ability to provide high-resolution three-dimensional datasets. It allows for assessment of vascular and valvular flow (a feature which is highly valuable in conditions such as aortic stenosis), quantification of shunts and accurate measurement of myocardial function. Data obtained from this modality can thus be visualised and reconstructed in any plane in order to assess complex cardiac anomalies [28]. Delineation of extra-cardiac anatomy (e.g. the great vessels) can be carried out with high spatial resolution: a feature that echocardiography alone cannot provide [5]. This provides a valid justification for the use of CMR in patients for whom clinical and echocardiographic data alone is insufficient in determining a diagnosis which could otherwise affect their ability to thrive [28].

Despite the numerous advantages CMR offers, there remain technical limitations which require careful consideration. In the absence of contrast or sedation, CMR carries minimal risk [28]. However, with the incorporation of general anaesthesia, gadolinium contrast or sedation, the risk profile for certain patients can change in CMR [28] Moreover, carrying out CMR requires multiple resources, the most important of which is the MR scanner which can be significantly costly and require expertise as well as adequate training to operate [28]. Paediatric CMR also poses diagnostic complexity due to the small size of the patients in proportion to the scanner. The paediatric heart also beats faster than in an adult patient, making it increasingly difficult to expedite image planning and optimise pulse sequences [28]

### Cardiac catheterisation

As of 1984, angiography at the time of cardiac catheterisation was used to diagnose VAS20. However, it was hypothesised that the use of the diagnostic modality may pose clinical harm to unstable infants and may result in haemodynamic instability [18, 29, 30]. In current practice, catheterisation is used for its therapeutic benefits rather than solely for diagnostic purposes [28, 30]. This procedure is mainly utilised by interventional cardiologists, specifically for balloon angioplasty of stenotic lesions, coarctation of aorta and valvuloplasty of stenotic aortic valves [23]. An example of one such therapeutic procedure which is routinely carried out in the cardiac catheter laboratory is aortic balloon valvuloplasty for aortic stenosis [27]. This is carried out using a transfemoral approach with access through both the femoral artery and vein. Both left and right heart catheterisation can be carried out for all patients [27, 28].

## Management

Management of aortic valve pathologies in paediatric patients can be categorised into medical, percutaneous and surgical. The latter is divided into valve repair or replacement procedures.

### Medical management

There is limited role for medication to treat congenital aortic valve pathologies. Vasodilators work by reducing afterload by improving stroke volume in severe AR. They are used in severe aortic valve insufficiency with LV dilation but normal LV systolic function [30]. Aortic valve regurgitation is managed according to severity, symptomatology, LV function and size. In mild cases with no change in cardiac size, no medical intervention is required, and patients are monitored with echocardiography every 12 months. Moderate-severe cases require surgical correction.

### Percutaneous repair

Valvuloplasty is often the initial treatment in paediatric patients with congenital aortic stenosis. And severe aortic valve insufficiency with symptoms and/or reduction in LV function. Fratz et al. [31] found that valvuloplasty is largely used as a palliative procedure with 59% post-procedure survival at 10 years. The main reason for repeat aortic valve surgery after valvuloplasty is the onset and severity of aortic regurgitation. The two types of valvuloplasty include balloon valvuloplasty (BAD) and surgical aortic valvuloplasty (SAV).

Hill et al. [32] conducted a meta-analysis comparing percutaneous with open aortic valve repair procedures across 20 studies of congenital aortic valve stenosis. Kaplan-Meier curves did not show any statistically significant difference in survival between the groups. Survival was 87% in the balloon valvuloplasty group compared to 90% in open valvotomy. Whilst there were no differences in long-term survival, there were significantly higher rates of reintervention following balloon valvuloplasty across infants and children subgroups.

d’Udekem et al.’s study [33] supports the superiority of valvotomy over valvuloplasty, concluding that patients undergoing balloon valvuloplasty similarly had a higher risk of regurgitation compared to open repair requiring reintervention. This may be due to the fact that valvuloplasty involves the breaking of the valve in the thinnest, weakest part of the valve leaflet. The onset of aortic regurgitation is also a common indication for replacement surgery in patients initially treated with repair. This is also the case in patients with congenital bicuspid valves [34].

### Surgical management

#### Aortic valve repair

Repair is usually attempted initially before replacement in tricuspid aortic valve pathologies. This is a preferred approach, particularly in younger patients, to delay the requirement of a valve replacement which is associated with further surgical intervention, limited graft duration and need for resizing.

Various techniques exist for aortic valve repair including commissurotomy, leaflet extension techniques, subcommissural aortic valve annuloplasty and patch reconstruction.

Aortic valve neocuspidalisation (Ozaki procedure) involves replacing the aortic valve leaflet with cut templates of autologous pericardium [35]. This was reported after a study involving 850 patients between 2007 and 2015 demonstrating acceptable performance with a cumulative incidence of aortic insufficiency of 7.3% at 10 years. Whilst the Ozaki procedure was initially invented for the adult population, Baird et al. [36] applied and adapted the procedure to a paediatric population. The adaptations included the need for aortic annulus and root size modification. This remains an option for patients with aortic regurgitation and stenosis and those who have aortic valve disease secondary to connective tissue disorders in whom a Ross procedure would not be recommended.

#### Aortic valve replacement

Replacement can occur using tissue (such as porcine or bovine) or mechanical valves as highlighted in Table 2.

**Table 2:** Summary of aortic valve replacement options

	Tissue prosthesis	Mechanical prosthesis	Ross procedure
Summary of procedure	Bovine or bioprosthesis	Mesh device	Pulmonary autograft
Risks	Requirement for second valve replacement	The valve can outgrow as the child grows requiring repeat surgery Anticoagulation	Requirement for second valve replacement
Association with poor outcomes	-	Younger age Presence of LV dysfunction Concomitant cardiac anomalies requiring surgery	-

Generally, the use of mechanical valves is reserved for children with connective tissue disorders and those in whom the native pulmonary valve is unsuitable for translocation to the aortic position for the Ross procedure. The procedure often involves enlargement of the annulus root to be able to insert the prostheses. There are various procedures enabling the insertion of the prostheses including Konno, Yamaguchi and Manouguian, as shown in Table 3. The Yamaguchi procedure is shown to be the safest as there is a reduced risk of damage to the septum or mitral leaflets [37].

**Table 3:** Summary of common mechanical aortic valve procedure techniques

	Yamaguchi procedure	Konno procedure	Manouguian procedure
Procedure	Incision only traverses the annulus without entering the RV or septum.	Incision of the ventricular septum	Incision extended to the anterior mitral leaflet
Risks	Aortic root dilation and aneurysm	Ventricular dysfunction or conduction abnormality	Mitral insufficiency

Tissue valves generally have early calcification and degeneration along with overall lower durability of the valve. They are also restricted in their use due to size constraints. For this reason, neonates are unlikely to be suitable to have tissue replacement. Tissue valves do not require anticoagulation thus offering this form of replacement an advantage when compared to mechanical valve substitutes.

The main risk associated with mechanical valves is thrombo-embolism and the need for long-term anticoagulation. This can impact lifestyle and introduce complications, particularly with hypercoagulable states such as pregnancy and peri-surgery. In young children and adolescents, compliance can also be an issue. Alsoufi et al.’s study [38] has shown that children with mechanical valve replacement have a 15-year survival of 75–88%. In the paediatric population, with patient growth, there is also an increased potential of the child to outgrow mechanical valves.

Lupinetti et al. [39] compared mechanical with bioprosthetic valve recipients undergoing aortic valve replacement and found the most common complication in the mechanical valve subgroup was the incidence of subvalvular stenosis. This may arise as the growth of the heart exceeds the capacity of the valve to function appropriately and cause pannus formation in the subvalvular area. This in turn increases the risk of reintervention.

Introduced in the 1990s, the Ross procedure involves replacing the aortic valve with a pulmonary autograft and the pulmonary valve with a pulmonary homograft. It remains an option for patients with various left ventricular outflow tract and aortic valve pathologies.

Patients with severe annular hypoplasia and complex LVOT obstruction can have a modified Ross procedure called Ross-Konno [39]. This involves an aorto-ventriculoplasty in addition to a Ross. The procedure also involves making an incision into the aortic annulus and creating a VSD which is later closed with a patch.

Replacement of the aortic valve with a pulmonary autograft as done in the Ross procedure can introduce concerns about autograft dilatation and subsequent aneurysms and aortic regurgitation. This is because of the incision across the annulus into the septum and insertion of a VSD patch. Ruzmetov et al. compared long-term outcomes of aortic valve replacements in 147 patients and found that the 10-year survival was highest in patients following the Ross procedure (98%) compared to tissue prosthesis (82%) and mechanical prosthesis (88%). Alsoufi et al. [39] also compared outcomes in a total of 346 patients (215 for Ross and 131 for mechanical prosthesis) and found significant postoperative mortality rates in the mechanical prosthesis arm compared to the Ross arm. Through competing risk analysis, after 16 years of valve replacement, there was a 20% mortality rate in the mechanical prosthesis subgroup; 25% of patients undergoing replacement required reintervention surgery and 55% of those undergoing valve replacement required no further treatment. Factors associated with increased mortality included mechanical prosthesis valves, younger age and smaller valve size. Karamlou et al. [40] found pulmonary autograft to have slower gradient progression and smaller left ventricular dimension.

#### Double valve replacement

Multiple valves are commonly affected by rheumatic heart disease, infective endocarditis and congenital heart disease including syndromic disease. 10% of patients with double valve disease have involvement of both aortic and mitral valve [41] necessitating replacement of both valves often in multi-step surgeries.

## Choice of procedure

The Ross procedure is currently the gold standard for aortic valve replacement surgery [41]. Studies have shown greater postoperative mortality in patients with poor preoperative clinical baseline. This includes patients with co-existent multiple valvular pathology, such as concomitant mitral valve disease and aortic arch disease. In such patients, a single-ventricle palliative approach may improve quality of life. This is also the case for neonates in whom valve replacement is limited due to multiple coexisting congenital cardiac anomalies and associated with greater surgical risks. Table 4 shows the various parameters in determining the rate of reintervention and replacement; intervention at less than 1 year of age and use of a cusp extension technique had statistically significant results for greater freedom from reintervention and replacement, thus offering an attractive choice of procedure in this age category.

**Table 4:** Predictors of freedom from aortic valve reintervention or replacement from d’Udekem et al.

	Freedom from reintervention (*p* value, HR)	Freedom from valve replacement (*p* value, HR)
Gender	0.948, 1.04	0.721, 0.81
Indication		
Aortic insufficiency	0.971, 0.98	0.865, 1.09
Aortic stenosis	0.806, 1.13	0.994, 1
Mixed stenosis/insufficiency	0.633, 0.66	0.748, 0.71
Concomitant cardiac procedure	0.256, 0.48	0.132, 0.31
Previous cardiac surgery	0.963, 1.03	0.82, 1.13
Age <1 year at time of surgery	0.048, 2.89	0.122, 2.37
Cusp extension technique	0.020, 3.34	0.011, 3.95

Tissue prosthesis may be appropriate in females of childbearing age for whom taking anticoagulation during pregnancy may pose a risk and also in children who may be noncompliant with anticoagulation.

Mechanical prosthesis may be more appropriate for patients with largely aortic regurgitation and dilated AV annulus.

Homografts are usually associated with a lower rate of operative mortality and so, an option in neonates or patients with invasive endocarditis is offered.

## Long-term results and outcomes

Despite the many advances in aortic valve surgery, children may still experience a significant reduction in their quality of life (QoL) due to problems in areas such as education, social and cognition [42, 43]. A study assessing young adults having undergone corrective cardiac surgery with a 10-year follow-up found them to have a reduced QoL, exercise capacity and physical activity than their age-matched healthy peers [44]. A 2014 cross-sectional survey used the PedsQL model to compare health-related quality of life outcomes (HRQOL) in children (8–12 years) and adolescents (13–18 years) with CHD against healthy controls. The PedsQL model is based upon the core dimensions of health (physical, emotional, social and school functioning) as defined by the World Health Organization. The study reported significantly lower HRQOL in children and adolescents with CHD requiring surgical or catheter-based intervention than healthy controls. The study also found that the recorded HRQOL in these children were similar to paediatric patients in other chronic disease populations [45].

Studies of developmental outcomes show that children with congenital heart disease (CHD) are at an increased risk of neurodevelopmental problems compared to healthy children, at all points of development throughout infancy to adolescence [46]. Brain MRI studies have revealed a pattern of delayed brain maturation and evidence of chronic diffuse white matter injury in foetuses with AVS with secondary HLHS. In addition, abnormal neurologic exams and microcephaly have also been noted in neonates with AVS with secondary HLHS [47–49]. A low-average to average IQ range in conjunction with mild deficits in a range of domains such as executive function and social cognition is common amongst children with wide-ranging diagnoses of CHD. The development of neurocognitive deficits is attributable to a number of factors including altered prenatal brain maturation, perioperative and postoperative events and comorbid genetic conditions [50]. The presence of such deficits predisposes children with CHD to requiring the assistance of special education services, resulting in a significant impact both upon themselves and their families [51, 52].

Although paediatric aortic valve repair has steadily garnered a greater interest, aortic valve replacement in children may be unavoidable [53]. A 2015 study compared the durability of aortic valve repair in children, namely aortic valve repair, AVR or Ross procedure. They concluded that freedom from surgical reintervention at the 6-year mark was 64, 100 and 51% for aortic valve repair, Ross and AVR groups respectively. The durability of aortic valve repair was found to be limited by the recurrence of aortic insufficiency and/or stenosis and so reoperation following the repair can be expected within a 7-year period [54]. Redo surgery in later life may be unavoidable in patients who have had aortic valve replacement as opposed to repair given durability of the valve in long-term complications. This inevitably affects quality of life given the reintervention procedures. There is also the problem of patient-prosthesis mismatch resulting in the patient requiring potentially multiple surgeries in the future to upgrade the size of the valve.

## Further research

Surgical repair of the paediatric aortic valve is an ever-developing area of research. The current gold-standard surgical procedure used in congenital aortic stenosis is the Ross procedure [55]. Although this procedure is well established, it is worth noting that there are some limitations associated, such as the need for expertise, extensive training and lifelong monitoring of patients [55]. There are also known complications which may arise as a result of carrying it out—development of regurgitation or stenosis in the pulmonary homograft 15–20 years post-procedure [55], cerebrovascular events, arrhythmias, haemorrhage and respiratory distress [38], to name a few. An emergent option which is currently being considered in paediatric patients is aortic valve neocuspidisation (AVNeo) [20], a procedure which involves the full removal of pathological aortic valve leaflets and individually replacing these with autologous pericardium [19]. A study was conducted by Ozaki et al. [34] in which 850 adult patients with several different aortic valve pathologies underwent an AVNeo procedure over a period of 8 years. The results showed excellent outcomes, with reduced cumulative incidence of reoperation and absence of recurrent moderate aortic regurgitation [20, 22]. The team subsequently performed the procedure on 10 paediatric patients who were deemed not suitable for the Ross procedure and the initial impression was positive [20]. It is currently questionable whether or not the artificial valve cusps will behave as well as they did in the adult cohort in a growing paediatric valve. It is also a point of research to improve valve repair viability to prevent the need for reoperation and in order to improve the overall quality of life in the paediatric population.

## Conclusion

Aortic valve abnormalities require early intervention. This is to prevent the onset and/or progression of complications and to improve the patient’s quality of life. Consequences of late intervention include the development of AVS, HLHS, infective endocarditis and various neurodevelopment problems. Treatment involves repair or replacement of the valve. Repair is often undertaken first in order to delay the need for valve replacement, tide the patient over until optimal timing for replacement and lengthen the lifespan of the graft. Although there are certain indications for the use of each subtype of valve repair and replacement, valvotomy is the more favoured approach to valve repair.

## Data Availability

None.

## Abbreviations

AVS: Aortic valve stenosis
AVA: Aortic valve atresia
LVOT: Left ventricular outflow tract
AS: Aortic stenosis
AVR: Aortic valve replacement
HLHS: Hypoplastic left heart syndrome
TTE: Transthoracic echocardiography
BAV: Bicuspid aortic valve
AVA: Aortic valve area
TAVR: Transcatheter aortic valve replacement
BAD: Balloon valvuloplasty
SAV: Surgical aortic valvuloplasty
VSD: Ventricular septal defect
LVOT: Left ventricular outflow obstruction

## Code availability

n/a
